# Inhibition studies on a panel of human carbonic anhydrases with *N*1-substituted secondary sulfonamides incorporating thiazolinone or imidazolone-indole tails

**DOI:** 10.1080/14756366.2018.1446432

**Published:** 2018-03-14

**Authors:** Fadi M. Awadallah, Silvia Bua, Walaa R. Mahmoud, Hossam H. Nada, Alessio Nocentini, Claudiu T. Supuran

**Affiliations:** a Pharmaceutical Chemistry Department, Faculty of Pharmacy, Cairo University, Cairo, Egypt;; b Department NEUROFARBA – Pharmaceutical and Nutraceutical Section, University of Firenze, Firenze, Italy;; c Pharmaceutical Chemistry Department, Faculty of Pharmacy, Badr University, Cairo, Egypt

**Keywords:** Carbonic anhydrase, sulfonamides, inhibitor, indole, zinc binding-group

## Abstract

Being the primary sulfonamide among the most efficient zinc binding group (ZBG) to design inhibitors for the metallo-enzymes carbonic anhydrases (CA, EC 4.2.1.1), herein, we propose an investigation on four physiologically important human (h) CAs (hCA I, II, IV, and IX) with *N*^1^-substituted secondary sulfonamides incorporating thiazolinone or imidazolone-indole tails. The effect of the functionalisation of the sulfonamide group with five different substitution patterns, namely acetyl, pyridine, thiazole, pyrimidine, and carbamimidoyl, was evaluated in relation to the inhibition profile of the corresponding primary sulfonamide analogues. With most of these latter being nanomolar inhibitors of all four considered isoforms, a totally counterproductive effect on the inhibition potency can be ascribed to *N*^1^-functionalisations of the ZBG primary sulfonamide structure with pyridine, thiazole, and pyrimidine moieties. On the other hand, incorporation of less hindered groups, such as sulfonylacetamides and sulfonylguanidines, maintained a certain degree of activity dependent on the tailing moiety, with K_I_s spanning in the low micromolar range.

## 1. Introduction

**Figure 1.**
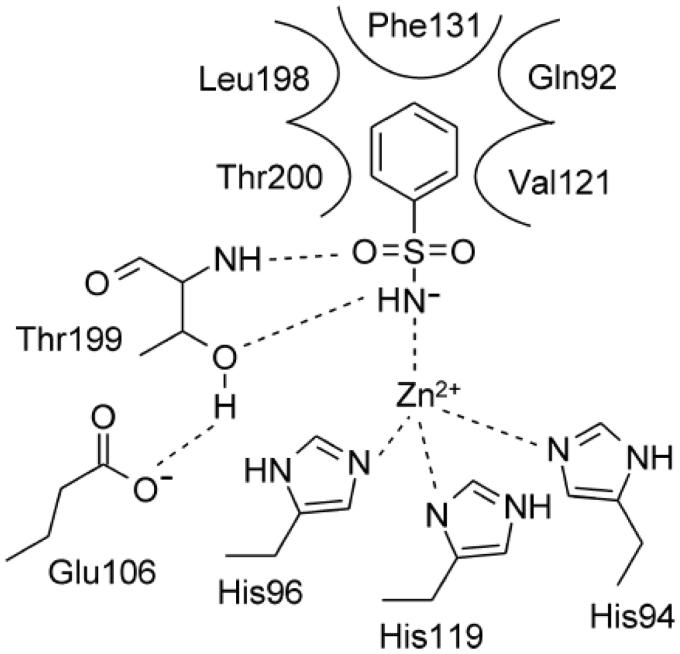
Schematic representation of the binding mode of benzenesulfonamide within the hCA II active site.

Primary sulfonamide is the most efficient zinc binding group (ZBG) to design inhibitors for the metallo-enzymes carbonic anhydrases (CA, EC 4.2.1.1), being its structural features ideal for the binding to the Zn^2+^ ion present at the bottom of the active site cavity and the residues nearby^1–3^. The negatively charged nitrogen of SO_2_NH^−^ coordinates the positively charged metal ion replacing the physiological zinc-bound nucleophile, with the proton on the coordinated nitrogen atom being at H-bond distance to Thr199 OG1 atom, which act as acceptor (Figure 1)^1^.

Benzenesulfonamides constitute the most common and best characterised class of CA inhibitors (CAIs)^3^. The presence of an aromatic/heteroaromatic scaffold bearing the sulfonamide group further stabilise the enzyme-ligand complex, by several Van der Waals interactions taking place with residues Gln92, Val121, Phe131, Leu198, and Thr200 (hCA II binding site (Figure 1))^1^^,^^3^.

All CAs found in humans belong to the α-class (α-CA) and are characterised as sixteen isoforms, which differ by molecular features, oligomeric arrangement, cellular localisation, distribution in organs and tissues, expression levels, and kinetic properties^1^^,^^2^. Abnormal levels or activities of most sixteen hCA isoforms have been often associated with different human diseases, making these isozymes of great interest for the design of inhibitors which selectively target specific isoforms, in order to reduce the overall side effects exhibited by most non-isoform selective CAIs clinically used up to now^1–4^. Most efforts to design isoform selective CAIs have been pursued by modulating the ring directly linked to the ZBG (ring approach)^3^^,^^5^ or appending different tails to the aromatic ring just mentioned (tail approach)^3^^,^^5–7^.

The previously accepted idea^8^ that only primary sulfonamides may act as effective inhibitors has been shown to be incorrect, but only few modifications were carried out on the sulfonamide moiety^1^a, in comparison to the high number of tails appended at the aromatic ring incorporating this ZGB^6^^,^^3^^,^^9^^,^^10^. The rational of this choice could be that of not losing the ideal features of primary sulfonamides, i.e. the negative charge on nitrogen and the presence of a proton on it, which are both important for the inhibition mechanism^1–3^. Conversely, it should be taken into account that *N*^1^-substitution with proper functional moieties could also elicit novel inhibition mechanisms^1^.

Secondary sulfonamides maintain the possibility to coordinate the Zn ion in the deprotonated form, as confirmed by X-ray crystallography. Di Fiore et al. demonstrated that *N*-hydroxy and *N*-methoxy sulfonamides are able to bind to the Zn(II) ion in the deprotonated form^11^. An analogous inhibition mechanism has been identified for the cyclic secondary sulfonamide saccharin and its derivatives^12^. Likewise, we previously reported that the incorporation of a nitro-group at the *N*^1^-atom of aromatic sulfonamide moieties does not deprive them of CA inhibitory efficacy, although the precise inhibitory mode has not been clarified yet^13^.

Tertiary sulfonamides lose their acid character, paving the way to a totally new inhibition mechanism. Carradori et al.^14^^,^^15^ recently synthesised different series of tertiary sulfonamides, namely probenecid and saccharin derivatives, obtaining interesting and selective inhibition profile. However, the inhibition mechanism with such compounds was not yet been elucidated. A plethora of series of benzenesulfonamide bearing indole or thiazolidinone moieties have also been reported so far and demonstrated to possess an effective inhibitory profile against a wide panel of hCAs^16–20^.

In an earlier investigation of some of us, a series of benzenesulfonamides linked to a chromone nucleus through thiazolinone and imidazolone spacers demonstrated excellent inhibitory activity against a panel of hCAs in a nanomolar range^21^. In continuation of the previous work, and with the aim to pursue the identification of new potent and selective CAIs, herein we explore the effect of the functionalisation of the sulfonamide group on the inhibitory properties by appending several moieties, namely acetyl, pyridine, thiazole, pyrimidine, and carbamimidoyl, at the *N*^1^-atom. A consistent series of imine-, thiazolinone-, and imidazolone benzenesulfonamides appending an indole ring instead of the chromone nucleus (from our earlier work) was thus obtained. All the synthesised derivatives were tested *in vitro* in order to evaluate their inhibition profiles against four physiologically important hCAs, i.e. hCA I, II, IV, and IX. Comparison of the inhibitory efficacy of primary sulfonamide derivatives with the *N*^1^-substituted analogues further strengthened the role of the sulfonamide in the pharmaceutical field related to CAs.

## Materials and methods

### Chemistry

Melting points were recorded on a Stuart SMP10 digital melting point apparatus and were uncorrected. Infrared (IR) spectra were recorded as KBr disks using a Shimadzu FT-IR 8400S infrared spectrophotometer. Mass spectral data are given as *m/z* (intensity %). ^1^H NMR spectra were recorded on either on a Varian Mercury VX-300 MHz spectrophotometer or Bruker AVANCE III Nano Bay 400 MHz FT-NMR spectrophotometer. ^13^C NMR spectra were run at 100 MHz in deuterated dimethylsulfoxide (DMSO-*d*_6_) on Bruker AVANCE III Nano Bay 400 MHz FT-NMR spectrophotometer. Chemical shifts are expressed in *δ* values (ppm) using the solvent peak as internal standard. All coupling constant (*J*) values are given in Hz. The abbreviations used are as follows: s, singlet; d, doublet; t, triplet; m, multiplet. Elemental microanalyses were carried out at the Regional Center for Mycology and Biotechnology, Al-Azhar University, Egypt. Reaction were routinely monitored by Thin Layer Chromatography (TLC) on silica gel precoated F254 Merck plates. Unless otherwise noted, all solvents and reagents were commercially available and used without further purification. Compounds **2**^22^, **4a–f**^23^^,^^24^, **6a–f**^23^^,^^24^, **7a**^25^, **8a,b**^26^^,^^27^ and **9a**^28^ were prepared according to the reported procedures.

### General procedure for the preparation of compounds 4a-f

A mixture of indole-3-carboxaldehyde **2** (1.45 g, 10 mmol) and the appropriate sulfonamide **3a–f** (10 mmol) were grinded and mixed together using a mortar and a pestle. The mixture was fused on a watch glass using drops of glacial acetic acid. The resulted paste was washed with ethanol/water mixture then crystallised from ethanol to afford compounds **4a–f**.

### 4-{[(1H-indol-3-yl)methylene]amino}benzenesulfonamide (4a)

Yellowish brown powder, (yield 60%), m.p. 180–182 °C; IR (KBr, ν cm^−1^): 3479–3244 (NHs), 1311 and 1149 (SO_2_); ^1^H NMR (DMSO-*d*_6_, 400 MHz) *δ* ppm: 5.80 (s, 2H, NH_2_, D_2_O exchangeable), 6.58 (d, 2H, Ar–H, *J* = 8.6), 6.88 (s, 1H, NH, D_2_O exchangeable), 7.21–7.26 (m, 2H, Ar–H), 7.45 (d, 2H, Ar–H, *J* = 8.6), 7.51 (d, 1H, Ar–H, *J* = 7.7), 8.09 (d, 1H, Ar–H, *J* =7.3), 8.28 (s, 1H, Ar–H), 9.93 (s, 1H, Ar–H); MS *m/z*: 299.0 [M]^+^; Anal. Calcd. for C_15_H_13_N_3_O_2_S (299.35): C, 60.18; H, 4.38; N, 14.04; Found C, 60.41; H, 4.28; N, 13.89.

### N-{[4-(((1H-indol-3-yl)methylene)amino)phenyl]sulfonyl}acetamide (4 b)

Off-white powder, (yield 70%), m.p. 170–172 °C; IR (KBr, ν cm^−1^): 3379–3200 (NHs), 1635 (C = O), 1334 and 1127 (SO_2_); ^1^H NMR (DMSO-*d*_6_, 400 MHz) *δ* ppm: 2.06 (s, 3H, CH_3_), 5.38 (s, 2H, 2NH, D_2_O exchangeable), 6.42–6.44 (m, 2H, Ar–H), 6.56 (d, 1H, Ar–H), 7.36 (d, 2H, Ar–H, *J* = 8.8), 7.52 (d, 2H, Ar–H, *J* = 8.8), 7.61 (d, 1H, Ar–H, *J* =8.8), 8.46 (s, 1H, Ar–H), 9.90 (s, 1H, Ar–H); ^13 ^C NMR (DMSO-*d*_6_) δ ppm: 27.1, 110.0, 112.2, 112.8, 121.0, 122.5, 123.5, 127.8, 128.4, 133.4, 150.6, 173.9 175.3; MS *m/z*: 341.42 [M]^+;^ Anal. Calcd. for C_17_H_15_N_3_O_3_S (341.38): C, 59.81; H, 4.43; N, 12.31; Found C, 59.63; H, 4.74; N, 12.52.

### 4-[((1H-indol-3-yl)methylene)amino)-N-(pyrimidin-2-yl)]benzenesulfonamide (4c)

Brown powder, (yield 68%), m.p. 217–219 °C; IR (KBr, ν cm^−1^): 3230–3147 (NHs), 1334 and 1126 (SO_2_); ^1^H NMR (DMSO-*d*_6_, 400 MHz) *δ* ppm: 5.68 (s, 1H, NH, D_2_O exchangeable), 6.54 (d, 2H, Ar–H, *J* = 8.6), 7.20–7.29 (m, 4H, Ar–H), 7.39 (d, 2H, Ar–H, *J* = 7.6), 7.51 (d, 1H, Ar–H, *J* = 7.6), 8.09 (d, 2H, Ar–H, *J* = 7.6), 8.29 (s, 1H, Ar–H), 9.86 (s, 1H, Ar–H), 12.13 (s, 1H, NH, D_2_O exchangeable); ^13 ^C NMR (DMSO-*d*_6_) *δ* ppm: 112.5, 112.8, 115.8, 118.6, 121.2, 122.5, 123.9, 124.5, 125.5, 130.2, 137.4, 138.9, 153.3, 158.6, 159.8, 185.4; MS *m/z*: 377.12 [M]^+^; Anal. Calcd. for C_19_H_19_N_5_O_2_S (377.42): C, 60.46; H, 4.01; N, 18.56; Found C, 60.29; H, 3.97; N, 18.64.

### 4-[((1H-indol-3-yl)methylene)amino)-N-(pyridin-2-yl)]benzenesulfonamide (4d)

Yellowish brown powder, (yield 55%), m.p. 144–146 °C; IR (KBr, ν cm^−1^): 3417–3207 (NHs), 1323 and 1126 (SO_2_); ^1^H NMR (DMSO-*d*_6_, 400 MHz) *δ* ppm: 5.91 (s, 1H, NH, D_2_O exchangeable), 6.53 (d, 1H, Ar–H, *J* = 9.6), 6.86 (t, 1H, Ar–H, *J* = 6.2), 7.04 (d, 2H, Ar–H, *J* = 8.4), 717–7.24 (m, 3H, Ar–H), 7.49 (d, 2H, Ar–H, *J* = 8), 7.62 (t, 1H, Ar–H, *J* = 8.8), 8.07 (d, 2H, Ar–H, *J* = 6.8), 8.26 (s, 1H, Ar–H), 9.91 (s, 1H, Ar–H), 12.10 (s, 1H, NH, D_2_O exchangeable); ^13 ^C NMR (DMSO-*d*_6_) *δ* ppm: 112.8, 118.6, 121.2, 122.5, 123.9, 124.5, 129.3, 137.4, 138.9, 153.1, 185.4; Anal. Calcd. for C_19_H_19_N_5_O_2_S (376.43): C, 63.81; H, 4.28; N, 14.88; Found C, 63.97; H, 4.19; N, 14.52.

### 4-[((1H-indol-3-yl)methylene)amino)-N-(thiazol-2-yl)]benzenesulfonamide (4e)

Yellowish brown powder, (yield 50%), m.p. 148–150 °C; IR (KBr, ν cm^−1^): 3367–3209 (NHs), 1334 and 1138 (SO_2_). ^1^H NMR (DMSO-*d*_6_, 400 MHz) *δ* ppm: 5.18 (s, 1H, NH, D_2_O exchangeable), 6.52 (t, 1H, Ar–H, *J* = 8), 7.15–7.26 (m, 2H, Ar–H), 7.35 (d, 1H, Ar–H, *J* = 8.8), 7.38 (d, 2H, Ar–H, *J* = 9.2), 7.49 (d, 2H, Ar–H, *J* = 7.6), 8.07 (d, 2H, Ar–H, *J* = 7.2), 8.26 (s, 1H, Ar–H), 9.91 (s, 1H, Ar–H), 12.10 (s, 1H, NH, D_2_O exchangeable); ^13 ^C NMR (DMSO-*d*_6_) *δ* ppm: 112.8, 112.9, 118.6, 121.2, 122.5, 123.9, 124.5, 128.0, 128.1, 137.4, 138.9, 152.6, 185.4; Anal. Calcd. for C_18_H_14_N_4_O_2_S_2_ (382.46): C, 56.53; H, 3.69; N, 14.65; Found C, 56.40; H, 3.62; N, 14.37.

### 4-{[(1H-indol-3-yl)methylene]amino}-N-carbamimidoylbenzenesulfonamide (4f)

Brown powder, (yield 68%), m.p. 154–156 °C; IR (KBr, ν cm^−1^): 3433–3332 (NHs), 1334 and 1126 (SO_2_); ^1^H NMR (DMSO-*d*_6_, 400 MHz) *δ* ppm: 5.65 (s, 3H, 3NHs, D_2_O exchangeable), 6.51 (d, 2H, Ar–H, *J* = 8.8), 7.19–7.24 (m, 2H, Ar–H), 7.35 (d, 1H, Ar–H, *J* = 8.4), 7.49 (d, 2H, Ar–H, *J* = 7.6), 8.06 (d, 1H, Ar–H, *J* = 8.0), 8.26 (s, 1H, Ar–H), 9.91 (s, 1H, Ar–H), 12.05 (s, 2H, 2NHs, D_2_O exchangeable); ^13 ^C NMR (DMSO-*d*_6_) *δ* ppm: 112.7, 112.8, 118.6, 121.2, 122.5, 123.9, 124.5, 127.6, 131.2, 137.4, 138.9, 151.8, 159.1, 185.4; Anal. Calcd. for C_16_H_15_N_5_O_2_S (341.39): C, 56.29; H, 4.43; N, 20.51; Found C, 56.01; H, 4.37; N, 20.79.

### General procedure for the preparation of compounds 7a–f

A mixture of indole-3-carboxaldehyde **2** (1.45 g, 10 mmol), the appropriate thiazolinone derivative **6a–f** (10 mmol) and fused sodium acetate (0.82 g, 10 mmol) in glacial acetic acid (50 ml) was refluxed for 12 h. The separated solid was filtered off while hot, washed with hot water and crystallised from DMF/H_2_O.

### 4-{[5-((1H-indol-3-yl)methylene)-4-oxo-4,5-dihydrothiazol-2-yl]amino}benzenesulfonamide (7a)

Yellowish brown powder, (yield 60%), m.p. 230–232 °C; IR (KBr, ν cm^−1^): 3360–3199 (NHs), 1674 (C = O), 1334 and 1151 (SO_2_); ^1^H NMR (DMSO-*d*_6_, 300 MHz) *δ* ppm: 7.14–7.25 (m, 4H, Ar–H), 7.34 (s, 1H, NH, D_2_O exchangeable), 7.48 (d, 2H, Ar–H, *J* = 7.5), 7.61 (s, 1H, Ar–H), 7.48 (d, 2H, Ar–H, *J* = 8.4), 7.94 (s, 1H, Ar–H), 11.89 (s, 2H, NH_2_, D_2_O exchangeable), 12.23 (s, 1H, NH, D_2_O exchangeable); ^13 ^C NMR (DMSO-*d*_6_) *δ* ppm: 115.1, 116.4, 119.8, 120.4, 124.7, 125.9, 126.9, 127.6, 129.6, 133.8, 137.8, 142.7, 144.0, 159.1, 169.7, 170.2; Anal. Calcd. for C_18_H_14_N_4_O_3_S_2_ (398.46): C, 54.26; H, 3.54; N, 14.06; Found C, 54.01; H, 3.75; N, 13.89.

### N-{[4-((5-((1H-indol-3-yl)methylene)-4-oxo-4,5-dihydrothiazol-2-yl)amino)phenyl]sulfonyl} acetamide (7 b)

Green powder, (yield 55%), m.p. 242–244 °C; IR (KBr, ν cm^−1^): 3371–3213 (NHs), 1701 and 1658 (2 C = Os), 1311 and 1149 (SO_2_); ^1^H NMR (DMSO-*d*_6_, 300 MHz) *δ* ppm: 1.94 (s, 3H, CH_3_), 7.18–7.27 (m, 4H, Ar–H), 7.49 (d, 1H, Ar–H, *J* = 9), 7.51 (s, 1H, NH, D_2_O exchangeable), 7.88 (d, 1H, Ar–H, *J* = 8.1), 8.07–8.09 (m, 3H, Ar–H), 8.26 (s, 1H, Ar–H), 12.10 (s, 2H, 2NHs, D_2_O exchangeable); Anal. Calcd. for C_20_H_16_N_4_O_4_S_2_ (440.50): C, 54.53; H, 3.66; N, 12.72; Found C, 54.62; H, 3.92; N, 12.93.

### 4-{[5-((1H-indol-3-yl)methylene)-4-oxo-4,5-dihydrothiazol-2-yl)amine]-N-(pyrimidin-2-yl)} benzenesulfonamide (7c)

Off-white powder, (yield 60%), m.p. 228–230 °C; IR (KBr, ν cm^−1^): 3317–3199 (NHs), 1681 (C = O), 1328 and 1150 (SO_2_); ^1^H NMR (DMSO-*d*_6_, 300 MHz) *δ* ppm: 7.03 (t, 2H, Ar–H, *J* = 4.8), 7.31–7.78 (m, 3H, Ar–H), 7.87 (d, 1H, *J* = 9), 7.89 (s, 2H, Ar–H), 8.07 (d, 3H, Ar–H, *J* = 8.7), 9.06 (d, 2H, Ar–H, *J* = 5.1), 10.84 (s, 3H, 3NHs, D_2_O exchangeable); Anal. Calcd. for C_22_H_16_N_6_O_3_S_2_ (476.53): C, 55.45; H, 3.38; N, 17.64; Found C, 55.37; H, 3.76; N, 17.41.

### 4-{[5-((1H-indol-3-yl)methylene)-4-oxo-4,5-dihydrothiazol-2-yl)amino]-N-(pyridin-2-yl)] benzenesulfonamide (7d)

Yellow powder, (yield 50%), m.p. 245–247 °C; IR (KBr, ν cm^−1^): 3350–3200 (NHs), 1697 (C = O), 1334 and 1138 (SO_2_); ^1^H NMR (DMSO-*d*_6_, 300 MHz) *δ* ppm: 7.37–7.45 (m, 5H, 4 Ar–H +1H, NH, D_2_O exchangeable), 7.63–7.45 (m, 4H, 2 Ar–H + 2H, 2NHs, D_2_O exchangeable), 8.13–8.17 (m, 3H, Ar–H), 8.34–8.43 (m, 3H, Ar–H), 8.87 (s, 2H, Ar–H); Anal. Calcd. for C_23_H_17_N_5_O_3_S_2_ (475.54): C, 58.09; H, 3.60; N, 14.73; Found C, 57.96; H, 3.42; N, 14.94.

### 4-{[(5-((1H-indol-3-yl)methylene)-4-oxo-4,5-dihydrothiazol-2-yl)amino]-N-(thiazol-2-yl)] benzenesulfonamide (7e)

Grey powder, (yield 60%), m.p. 218–220 °C; IR (KBr, ν cm^−1^): 3327–3207 (NHs), 1687 (C = O), 1317 and 1153 (SO_2_); ^1^H NMR (DMSO-*d*_6_, 300 MHz) *δ* ppm: 6.80–6.83 (m, 2H, Ar–H), 7.18–7.27 (m, 4H, Ar–H), 7.48 (t, 1H, Ar–H, *J* = 7.2), 7.81 (d, 2H, Ar–H, *J* = 9), 8.07 (d, 1H, Ar–H, *J* = 7,2), 8.26 (s, 1H, Ar–H), 8.27 (s, 1H, Ar–H), 11.95 (s, 1H, NH, D_2_O exchangeable), 12.10 (s, 2H, 2NHs, D_2_O exchangeable); Anal. Calcd. for C_21_H_15_N_5_O_3_S_3_ (481.57): C, 52.38; H, 3.14; N, 14.54; Found C, 52.53; H, 3.33; N, 14.71.

### 4-{[5-((1H-indol-3-yl)methylene)-4-oxo-4,5-dihydrothiazol-2-yl]amino}-N-carbamimidoyl benzenesulfonamide (7f)

Brown powder, (yield 65%), m.p. 227–229 °C; IR (KBr, ν cm^−1^): 3433–3209 (NHs), 1627 (C = O), 1334 and 1126 (SO_2_); ^1^H NMR (DMSO-*d*_6_, 300 MHz) *δ* ppm: 6.88 (s, 1H, Ar–H), 7.16–7.27 (m, 4H, Ar–H), 7.48 (t, 1H, Ar–H, *J* = 6.3), 7.89 (d, 2H, Ar–H, *J* = 8.7), 8.08 (d, 1H, Ar–H, *J* = 7.2), 8.27 (s, 1H, Ar–H), 11.91 (s, 3H, NHs, D_2_O exchangeable), 12.11 (s, 3H, NHs, D_2_O exchangeable); ^13 ^C NMR (DMSO-*d*_6_) *δ* ppm: 112.8, 114.4, 118.6, 118.7, 121.2, 122.5, 123.0, 123.9, 124.5, 127.2, 128.6, 136.7, 137.5, 138.9, 140.9, 170.9, 185.4; Anal. Calcd. for C_19_H_16_N_6_O_3_S_2_ (440.50): C, 51.81; H, 3.66; N, 19.08; Found C, 52.13; H, 3.90; N, 19.23.

### 4-[(1-Acetyl-1H-indol-3-yl)methylene]-2–(4-methoxyphenyl)oxazol-5(4H)-one (9 b)

A mixture of indole-3-carboxaldehyde **2** (1.45 g, 10 mmol), *N*-(4-methoxybenzoyl)glycine **8 b** (2.09 gm, 10 mmol) and fused sodium acetate (0.5 g, 6 mmol) in acetic anhydride (20 ml) was heated in a boiling water bath for 5 h. The mixture was cooled and ethanol (20 ml) was added slowly and allowed to stand overnight in the refrigerator. The crystalline product was filtered off, washed with hot water and recrystallised from benzene to give compound **9 b**.

Buff powder, (yield 60%), m.p. 149–151 °C; IR (KBr, ν cm^−1^): 1743 and 1689 (2 C = Os); ^1^H NMR (DMSO-*d*_6_, 400 MHz) *δ* ppm: 1.91 (s, 3H, CH_3_), 3.88 (s, 3H, OCH_3_), 7.17 (d, 2H, Ar–H, *J* = 8), 7.39–7.46 (m, 2H, Ar–H), 7.50 (s, 1H, Ar–H), 8.07 (d, 2H, Ar–H, *J* = 8), 8.35 (d, 2H, Ar–H, *J* = 8), 8.79 (s, 1H, Ar–H); Anal. Calcd. for C_21_H_16_N_2_O_4_ (360.36): C, 69.99; H, 4.48; N, 7.77; Found C, 70.23; H, 4.65; N, 7.91.

### General procedure for the preparation of compounds 10a–l

An equimolar amount of compound **9a,b** (10 mmol) and the appropriate sulfonamide **3a–f** (10 mmol) in glacial acetic acid (20 ml) containing freshly fused sodium acetate (0.03 g, 0.36 mmol) was heated in a boiling water bath with constant stirring for 10–14 h. The separated solid was filtered off, washed with water, and crystallised from DMF/water to give compounds **10a–l**.

### 4-[4-((1-Acetyl-1H-indol-3-yl)methylene)-5-oxo-2-phenyl-4,5-dihydro-1H-imidazol-1-yl]benzenesulfonamide (10a)

Grey powder, (yield 60%), m.p. 240–242 °C; IR (KBr, ν cm^−1^): 3371, 3305 (NH_2_), 1708 and 1650 (2 C = Os), 1338 and 1157 (SO_2_); ^1^H NMR (DMSO-*d*_6_, 300 MHz) *δ* ppm: 1.90 (s, 3H, CH_3_), 7.26 (s, 2H, NH_2_, D_2_O exchangeable), 7.31–7.42 (m, 2H, Ar–H), 7.45 (s, 1H, Ar–H), 7.51–7.61 (m, 3H, Ar–H), 7.79 (d, 1H, Ar–H, *J* = 9), 7.87 (d, 2H, Ar–H, *J* = 6.9), 7.93 (d, 1H, Ar–H, *J* = 9), 8.06 (d, 2H, Ar–H, *J* = 7.2), 8.16 (s, 1H, Ar–H), 8.33 (d, 2H, Ar–H, *J* = 6.9); ^13 ^C NMR (DMSO-*d*_6_) *δ* ppm: 24.1, 115.1, 116.4, 119.9, 120.4, 124.7, 125.9, 126.9, 127.6, 128.6, 128.9, 129.1, 131.2 132.3, 133.6, 137.5 138.9, 142.7 144.0, 168.4, 169.7, 170.2; Anal. Calcd. for C_26_H_20_N_4_O_4_S (484.53): C, 64.45; H, 4.16; N, 11.56; Found C, 64.18; H, 4.19; N, 11.48.

### N-[(4–(4-((1-acetyl-1H-indol-3-yl)methylene)-5-oxo-2-phenyl-4,5-dihydro-1H-imidazol-1-yl)phenyl)sulfonyl]acetamide (10 b)

Brown powder, (yield 55%), m.p. 237–239 °C; IR (KBr, ν cm^−1^): 3390 (NH), 1730, 1701 and 1635 (3 C = Os), 1327 and 1165 (SO_2_); ^1^H NMR (DMSO-*d*_6_, 300 MHz) *δ* ppm: 1.91 (s, 3H, CH_3_), 1.92 (s, 3H, CH_3_), 7.15–7.26 (m, 2H, Ar–H), 7.31–7.73 (m, 3H, Ar–H), 7.88 (d, 2H, Ar–H, *J* = 8.7), 7.93–7.99 (m, 2H, Ar–H), 8.05 (d, 2H, Ar–H, *J* = 6.9), 8.18 (s, 1H, Ar–H), 8.36 (d, 2H, Ar–H, *J* = 6.9), 8.81 (s, 1H, Ar–H), 12.51 (s, 1H, NH, D_2_O exchangeable); ^13 ^C NMR (DMSO-*d*_6_) *δ* ppm: 23.6, 24.1, 115.0, 116.3, 119.9, 124.2, 124.7, 125.9, 127.7, 128.3, 128.5, 128.8, 129.1, 129.3, 129.6, 131.1, 132.0, 132.3, 133.8, 135.0, 144.4, 164.9, 169.1, 169.7; Anal. Calcd. for C_28_H_22_N_4_O_5_S (526.56): C, 63.87; H, 4.21; N, 10.64; Found C, 63.81; H, 4.27; N, 10.97.

### 4-[4-((1-Acetyl-1H-indol-3-yl)methylene)-5-oxo-2-phenyl-4,5-dihydro-1H-imidazol-1-yl]-N-(pyrimidin-2-yl)benzenesulfonamide (10c)

Brownish red powder, (yield 55%), m.p. 248–250 °C; IR (KBr, ν cm^−1^): 3383 (NH), 1689 and 1643 (2 C = Os), 1323 and 1153 (SO_2_); ^1^H NMR (DMSO-*d*_6_, 400 MHz) δ ppm: 1.96 (s, 3H, CH_3_), 7.06 (t, 1H, Ar–H, *J* = 4.2), 7.37–7.48 (m, 3H, Ar–H), 7.59 (t, 2H, Ar–H, *J* = 7.3), 7.65 (d, 2H, Ar–H, *J* = 6.8), 7.92 (d, 2H, Ar–H, *J* = 7.8), 8.00 (s, 1H, Ar–H), 8.11 (d, 2H, Ar–H, *J* = 7.2), 8.23 (s, 1H, Ar–H), 8.39 (d, 2H, Ar–H, *J* = 8), 8.54 (d, 2H, Ar–H, J = 4.6), 11.91 (s, 1H, NH, D_2_O exchangeable); ^13 ^C NMR (DMSO-*d*_6_) *δ* ppm: 24.1, 115.0, 116.3, 119.7, 119.9, 124.2, 125.8, 127.7, 128.3, 128.5, 128.8, 129.0, 129.7, 131.2, 132.3, 133.8, 134.9, 144.4, 158,7, 164.8, 164.8, 169.7; Anal. Calcd. for C_30_H_22_N_6_O_4_S (562.60): C, 64.05; H, 3.94; N, 14.94; Found; C, 64.39; H, 4.20; N, 15.21.

### 4-[4-((1-Acetyl-1H-indol-3-yl)methylene)-5-oxo-2-phenyl-4,5-dihydro-1H-imidazol-1-y])-N-(pyridin-2-yl)benzenesulfonamidee (10d)

Yellow powder, (yield 60%), m.p. 248–250 °C; IR (KBr, ν cm^−1^): 3325 (NH), 1730 and 1631 (2 C = Os), 1327 and 1136 (SO_2_); ^1^H NMR (DMSO-*d*_6_, 300 MHz) *δ* ppm: 2.05 (s, 3H, CH_3_), 6.86 (t, 1H, Ar–H, *J* = 6.3), 7.11 (d, 1H, Ar–H, *J* = 8.7), 7.18–7.28 (m, 5H, Ar–H), 7.50 (d, 1H, Ar–H, *J* = 6.6), 7.65–7.69 (m, 4H, Ar–H), 7.70 (s, 1H, Ar–H), 7.79 (d, 2H, Ar–H, *J* = 8.7), 8.01 (s, 1H, Ar–H), 8.08 (d, 2H, Ar–H, *J* = 7.2), 8.28 (d, 1H, Ar–H, *J* = 6.6), 12.10 (s, 1H, NH, D_2_O exchangeable); Anal. Calcd. for C_31_H_23_N_5_O_4_S (561.61): C, 66.30; H, 4.13; N, 12.47; Found C, 66.23; H, 4.38; N, 12.68.

### 4-[4-((1-Acetyl-1H-indol-3-yl)methylene)-5-oxo-2-phenyl-4,5-dihydro-1H-imidazol-1-yl]-N-(thiazol-2-yl)benzenesulfonamide (10e)

Green powder, (yield 65%), m.p. 265–267 °C; IR (KBr, ν cm^−1^): 3251 (NH), 1703 and 1627 (2 C = Os), 1328 and 1147 (SO_2_); ^1^H NMR (DMSO-*d*_6_, 300 MHz) *δ* ppm: 1.89 (s, 3H, CH_3_), 6.80–6.87 (m, 2H, Ar–H), 7.23–7.37 (m, 2H, Ar–H), 7.42 (t, 1H, Ar–H, *J* = 6.9), 7.53 (t, 2H, Ar–H, *J* = 7.3), 7.77 (d, 2H, Ar–H, *J* = 8.7), 7.85–7.80 (m, 2H, Ar–H), 7.92 (s, 1H, Ar–H), 8.05 (d, 2H, *J* = 6.9), 8.16 (s, 1H, Ar–H), 8.33 (d, 2H, Ar–H, *J* = 8.1), 12.72 (s, 1H, NH, D_2_O exchangeable); ^13 ^C NMR (DMSO-*d*_6_) *δ* ppm: 24.3, 115.1, 116.4, 120.3, 123.9, 125.8, 126.8, 127.7, 128.9, 129.0, 129.7, 131.2, 133.9, 134.9, 137.2, 139.0, 142.2, 144.5, 158.6, 159.2, 168.9, 170.1; Anal. Calcd. for C_29_H_21_N_5_O_4_S_2_ (567.64): C, 61.36; H, 3.73; N, 12.34; Found C, 61.08; H, 3.44; N, 12.59.

### 4-[4-((1-Acetyl-1H-indol-3-yl)methylene)-5-oxo-2-phenyl-4,5-dihydro-1H-imidazol-1-yl]-N-carbamimidoylbenzenesulfonamide (10f)

Brown powder, (yield 65%), m.p. 252–254 °C; IR (KBr, ν cm^−1^): 3217 (NH), 1708 and 1635 (2 C = Os), 1315 and 1138 (SO_2_); ^1^H NMR (DMSO-*d*_6_, 300 MHz) *δ* ppm: 1.90 (s, 3H, CH_3_), 7.14–7.25 (m, 4H, Ar–H), 7.34 (s, 2H, NH_2_, D_2_O exchangeable), 7.61 (s, 1H, Ar–H), 7.47–7.61 (m, 5H, Ar–H), 7.83–7.94 (m, 5H, Ar–H), 11.89 (s, 2H, 2NHs, D_2_O exchangeable); ^13 ^C NMR (DMSO-d_6_) *δ* ppm: 24.4, 108.5, 115.0, 116.6, 119.8, 120.4, 124.6, 125.8, 127.2, 127.6, 128.5, 129.0, 129.7, 131.2, 132.3, 134.9, 135.7, 136.8, 142.3, 143.0, 164.9, 168.9, 169.7; Anal. Calcd. for C_27_H_22_N_6_O_4_S (526.57): C, 61.59; H, 4.21; N, 15.96; Found C, 61.31; H, 4.50; 16.04.

### 4-[4-((1-Acetyl-1H-indol-3-yl)methylene)-2–(4-methoxyphenyl)-5-oxo-4,5-dihydro-1H-imidazol-1-yl]benzenesulfonamide (10 g)

Off-white powder, (yield 70%), m.p. 226–228 °C; IR (KBr, ν cm^−1^): 3479, 3375 (NH_2_), 1627 and 1597 (2 C = Os), 1315 and 1145 (SO_2_); ^1^H NMR (DMSO-*d*_6_, 400 MHz) *δ* ppm: 2.08 (s, 3H, CH_3)_ , 3.80 (s, 3H, OCH_3_), 5.77 (s, 2H, NH_2_, D_2_O exchangeable), 7.5–6.85 (m, 3H, Ar–H), 6.85 (s, 1H, Ar–H), 6.99 (d, 1H, *J* = 7.2), 7.17–7.26 (m, 3H, Ar–H), 7.42 (d, 1H, Ar–H, *J* = 6.8), 7.86 (d, 2H, Ar–H, *J* = 7.2), 8.07 (d, 2H, Ar–H, *J* = 8.4), 8.26 (s, 1H, Ar–H); ^13 ^C NMR (DMSO-d_6_) *δ* ppm: 24.5, 55.7, 112.8, 113.9, 114.2, 118.9, 124.0, 127.0, 127.3, 127.8, 129.3, 133.0, 138.4, 140.9, 142.7, 152.4, 161.8, 162.9, 165.6, 169.5, 173.1, 173.9; Anal. Calcd. for C_27_H_22_N_4_O_5_S (514.55): C, 63.02; H, 4.31; N, 10.89; Found C, 63.09; H, 4.64; N, 11.07.

### N-[(4–(4-((1-acetyl-1H-indol-3-yl)methylene)-2–(4-methoxyphenyl)-5-oxo-4,5-dihydro-1H-imidazol-1-yl)phenyl)sulfonyl]acetamide (10 h)

Brown powder, (yield 70%), m.p. 234–236 °C; IR (KBr, ν cm^−1^): 3367 (NH), 1685 (br.) and 1604 (3 C = Os), 1330 and 1168 (SO_2_); ^1^H NMR (DMSO-*d*_6_, 400 MHz) *δ* ppm: 2.02 (s, 3H, CH_3_), 2.06 (s, 3H, CH_3_), 3.80 (s, 3H, OCH_3_), 7.21–7.33 (m, 3H, Ar–H), 7.42–7.44 (m, 2H, Ar–H), 7.51 (d, 1H, Ar–H, *J* = 8.8), 7.61 (d, 2H, Ar–H, *J* = 8.8), 7.72 (s, 1H, Ar–H), 7.84 (s, 1H, Ar–H), 7.85 (d, 2H, Ar–H, *J* = 7.2), 7.89 (d, 2H, Ar–H, *J* = 6.8), 10.36 (s, 1H, NH, D_2_O exchangeable); ^13 ^C NMR (DMSO-*d*_6_) *δ* ppm: 22.0, 24.5, 55.7, 112.9, 113.5, 113.8, 118.2, 118.5, 118.9, 121.2, 122.5, 123.8, 125.9, 127.1, 128.0, 131.3, 131.6, 138.4, 142.6, 162.5, 168.4, 169.5, 173.1, 185.5; Anal. Calcd. for C_29_H_24_N_4_O_6_S (556.59): C, 62.58; H, 4.35; N, 10.07; Found C, 62.24; H, 4.67; N, 10.03.

### 4-[4-((1-Acetyl-1H-indol-3-yl)methylene)-2–(4-methoxyphenyl)-5-oxo-4,5-dihydro-1H-imidazol-1-yl]-N-(pyrimidin-2-yl)benzenesulfonamide (10i)

Off-white powder, (yield 65%), m.p. 178–180 °C; IR (KBr, ν cm^−1^): 3356 (NH), 1651 and 1597 (2 C = Os), 1327 and 1153 (SO_2_); ^1^H NMR (DMSO-*d*_6_, 400 MHz) *δ* ppm: 2.07 (s, 3H, CH_3_), 3.81 (s, 3H, OCH_3_), 6.00 (s, 1H, NH, D_2_O exchangeable), 6.56 (d, 2H, Ar–H, *J* = 8.8), 6.99–7.03 (m, 3H, Ar–H), 7.61 (d, 2H, Ar–H, *J* = 8.7), 7.73 (d, 2H, Ar–H, *J* = 8.8), 7.84 (d, 2H, Ar–H, *J* = 8.8), 7.89 (d, 2H, Ar–H, *J* = 8.8), 7.95 (s, 1H, Ar–H), 8.25 (s, 1H, Ar–H), 8.47 (d, 2H, Ar–H, *J* = 8.8); Anal. Calcd. for C_31_H_24_N_6_O_5_S (592.62): C, 62.83; H, 4.08; N, 14.18; Found; C, 63.17; H, 4.40; N, 14.56.

### 4-[4-((1-Acetyl-1H-indol-3-yl)methylene)-2–(4-methoxyphenyl)-5-oxo-4,5-dihydro-1H-imidazol-1-yl]-N-(pyridin-2-yl)benzenesulfonamide (10j)

Dark brown powder, (yield 75%), m.p. 198–200 °C; IR (KBr, ν cm^−1^): 3244 (NH), 1660 and 1635 (2 C = Os), 1320 and 1126 (SO_2_); ^1^H NMR (DMSO-*d*_6_, 400 MHz) *δ* ppm: 2.04 (s, 3H, CH_3_), 3.80 (s, 3H, OCH_3_), 6.51–6.54 (m, 3H, Ar–H), 6.58–6.98 (m, 3H, Ar–H), 7.04 (t, 1H, Ar–H, *J* =10.6), 7.11–7.26 (m, 2H, Ar–H), 7.50 (d, 2H, Ar–H, *J* = 8.8), 7.71–7.64 (m, 4H, Ar–H), 8.07 (d, 2H, Ar–H, *J* = 8.4), 8.25 (s, 1H, Ar–H), 11.01 (s, 1H, NH, D_2_O exchangeable); Anal. Calcd. for C_32_H_24_N_5_O_5_S (591.64): C, 64.96; H, 4.26; N, 11.84; Found C, 65.19; H, 4.62; N, 11.88.

### 4-[4-((1-Acetyl-1H-indol-3-yl)methylene)-2–(4-methoxyphenyl)-5-oxo-4,5-dihydro-1H-imidazol-1-yl]-N-(thiazol-2-yl)benzenesulfonamide (10k)

Light brown powder, (yield 65%), m.p. 206–208 °C; IR (KBr, ν cm^−1^): 3294 (NH), 1670 and 1639 (2 C = Os), 1330 and 1145 (SO_2_); ^1^H NMR (DMSO-*d*_6_, 400 MHz) *δ* ppm: 2.06 (s, 3H, CH_3_), 3.80 (s, 3H, OCH_3_), 5.80 (d, 1H, Ar–H, *J* = 4.8), 6.49–6.55 (m, 3H, Ar–H), 6.72 (d, 1H, Ar–H, *J* = 4.4), 6.95–7.00 (m, 2H, Ar–H), 7.18 (d, 1H, Ar–H, *J* = 6.4), 7.35 (d, 1H, Ar–H, *J* = 8.4), 7.37 (s, 1H, Ar–H), 7.41 (d, 2H, Ar–H, *J* = 8.8), 7.73 (s, 1H, Ar–H), 7.55 (s, 1H, Ar–H), 7.87 (d, 2H, Ar–H, *J* = 8.8), 11.15 (s, 1H, NH, D_2_O exchangeable); Anal. Calcd. for C_30_H_23_N_5_O_5_S_2_ (597.66): C, 60.29; H, 3.88; N, 11.72; Found C, 60.13; H, 4.20; N, 11.70.

### 4-[4-((1-Acetyl-1H-indol-3-yl)methylene)-2–(4-methoxyphenyl)-5-oxo-4,5-dihydro-1H-imidazol-1-yl]-N-carbamimidoylbenzenesulfonamide (10 l)

Brown powder, (yield 70%), m.p. 222–224 °C; IR (KBr, ν cm^−1^): 3433–3213 (NHs), 1635 and 1608 (2 C = Os), 1300 and 1126 (SO_2_); ^1^H NMR (DMSO-*d*_6_, 400 MHZ) *δ* ppm: 1.90 (s, 3H, CH_3_), 3.80 (s, 3H, OCH_3_), 5.68 (s, 3H, NHs, D_2_O exchangeable), 6.54 (d, 2H, Ar–H*, J* = 8.6), 7.02 (d, 1H, Ar–H, *J* = 8.8), 7.20–7.28 (m, 2H, Ar–H), 7.39 (d, 2H, Ar–H, *J* = 8.6), 7.51 (d, 2H, Ar–H, *J* = 7.6), 7.67 (s, 1H, Ar–H), 7.90 (d, 1H, Ar–H, *J* = 8.8), 8.10 (d, 2H, Ar–H, *J* = 7.2), 8.29 (s, 1H, Ar–H), 12.13 (s, 1H, NH, D_2_O exchangeable); Anal. Calcd. for C_28_H_24_N_6_O_5_S (556.59): C, 60.42; H, 4.35; N, 15.10; Found C, 60.72; H, 4.62; N, 15.39.

### Carbonic anhydrase inhibition assay

An applied photophysics stopped-flow instrument has been used for assaying the CA catalysed CO_2_ hydration activity^29^. Phenol red (at a concentration of 0.2 mM) has been used as indicator, working at the absorbance maximum of 557 nm, with 20 Mm Hepes (pH 7.5) as buffer, and 20 mM Na_2_SO_4_ (for maintaining constant the ionic strength), following the initial rates of the CA-catalysed CO_2_ hydration reaction for a period of 10–100 s. The CO_2_ concentrations ranged from 1.7 to 17 mM for the determination of the kinetic parameters and inhibition constants. For each inhibitor at least six traces of the initial 5–10% of the reaction have been used for determining the initial rate. The uncatalysed rates were determine d in the same manner and subtracted from the total observed rates. Stock solutions of inhibitor (0.1 mM) were prepared in distilled-deionised water and dilutions up to 0.01 nM were done thereafter with the assay buffer. Inhibitor and enzyme solutions were preincubated together for 15 min at room temperature prior to assay, in order to allow for the formation of the E–I complex. The inhibition constants were obtained by non-linear least-squares methods using PRISM 3 and the Cheng–Prusoff equation, as reported earlier^30–33^, and represent the mean from at least three different determinations. All CA isoforms were recombinant ones obtained in-house as reported earlier^34–36^.

## Results and discussion

### Chemistry

**Scheme 1.**
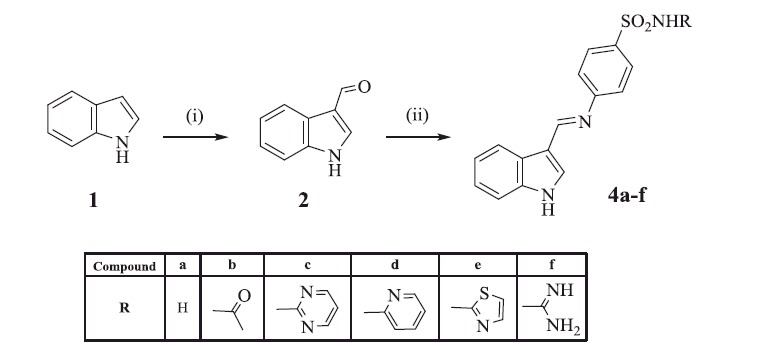
Synthesis of compounds **4a–f.** Reagents and reaction conditions: (i) Phosphorous oxychloride, DMF, 5 °C; (ii) glacial acetic acid.

**Scheme 2.**
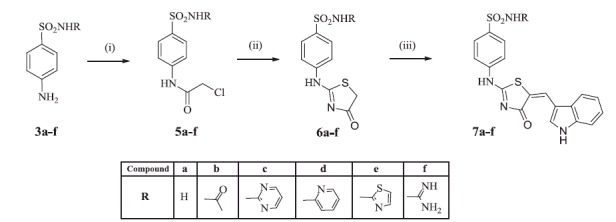
Synthesis of compounds **7a–f.** Reagents and reaction conditions: (i) Chloroacetyl chloride, DMF, rt; (ii) Ammonium thiocyanate, absolute alcohol; (iii) Indole-3-carboxaldehyde **2**, fused sodium acetate, glacial acetic acid, reflux.

**Scheme 3.**
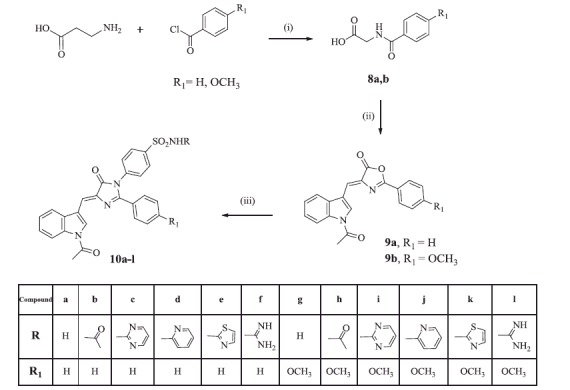
Synthesis of compounds **10a–l.** Reagents and reaction conditions: (i) Glycine, 10% sodium hydroxide, ice bath, (0 °C); (ii) Indole-3-carboxaldehyde **2**, acetic anhydride, fused sodium acetate, (100 °C); (iii) The appropriate sulfonamide **3a–f**, glacial acetic acid, fused sodium acetate, (100 °C).

The target compounds **4a–f**, **7a–f,** and **10a–l** were synthesised as illustrated in Schemes 1–3. The key stating material, indole-3-carboxaldehyde **2**, was prepared via Vilsemier-Hack formylation reaction, as reported in literature^22^. IR spectrum of compound **2** revealed the presence of (NH) stretching band at 3363 cm^−1^ along with a (C = O) stretching band at 1633 cm^−1^. In addition, ^1^H NMR showed a singlet signal at 9.94 ppm attributed to the proton of the (CH = O) group and a D_2_O exchangeable signal of the (NH) proton at 12.15 ppm. Schiff’s bases **4a–f** were obtained via the condensation of the aldehyde compound **2** with different sulfonamide derivatives **3a–f**. The reported method for the preparation of these compounds failed to give the expected compounds, and in some derivatives a very poor yield was obtained^37^. Searching for an alternative method, Naqvi et al.^38^ reported an energy efficient greener methodology for the preparation of Schiff’s bases involving a mechano-chemical solvent-free procedure. Applying this new methodology afforded compounds **4a–f** in good yields and high purity in a short reaction time. IR spectra of compounds **4a–f** were characterised by the disappearance of the (C = O) stretching band of the aldehyde **2** and broadening of the (NH) stretching band at 3350–3200 cm^−1^ due to the additional (NH) groups. Also, two characteristic stretching bands corresponding to the (SO_2_) group were identified at 1334–1311 and 1149–1126 cm^−1^. ^1^H NMR of compounds **4a–f** displayed the presence of sharp singlet signal at 9.86–9.93 ppm attributed to the azomethine proton (CH = N) in addition to the (NH) singlet signals, all disappeared by D_2_O exchange. As for compound **4 b**; a singlet signal at 2.06 ppm integrated for the three protons of the (CH_3_) group in ^1^H NMR spectrum and a signal at 27.1 ppm in ^13^C NMR spectrum confirmed its structure. In addition, ^13^C NMR of compounds **4c–f** adopted the expected pattern of their carbon content. Mass spectra of compounds **4a–c** gave *m/z* at 299.0, 341.42 and 377.42 corresponding to their molecular weights, respectively.

The thiazolinone derivatives **7a–f** were synthesised according to Scheme 2. The appropriate sulfonamide derivatives **3a–f** were reacted with chloroacetyl chloride at r.t. in DMF to give the intermediate chloroacetyl derivatives **5a–f** which were then intramolecularly cyclised by reflux with ammonium thiocyanate in ethanol, to obtain the thiazolinone compounds **6a–f**^23^^,^^24^. Knoevenagel condensation of the aldehyde **2** with the active methylene group in compounds **6a–f** afforded the newly synthesised compounds **7a–f**. IR spectra of compounds **7a–f** were consistent with their proposed structures where broad stretching bands appeared at 3433–3199 cm^−1^ corresponding to the (NH) groups. The (C = O) band of the thiazolinone ring appeared at 1697–1627 cm^−1^ with an additional (C = O) band at 1701 cm^−1^ in compound **7 b.** The two (SO_2_) stretching bands of all derivatives of the series were displayed at 1334–1311 and 1151–1126 cm^−1^. Furthermore, compounds **7a–f** showed characteristic ^1^H NMR signal in the region 7.78–8.87 ppm corresponding to the alkene (CH=) proton accompanied with the absence of the singlet signal corresponding to the aliphatic (CH_2_) protons with the usual pattern of the protons of both indole and benzene sulfonamide rings. The three protons of the acetamide group of compound **7 b** appeared as singlet signal at 1.94 ppm. ^13^C NMR spectra of compounds **7a** and **7f** were consistent with their carbon skeleton.

Scheme 3 illustrated the synthesis of the imidazolinone derivatives **10a–l**. The oxazolone intermediates **9a**^28^ and **9 b** were synthesised by Erlenmeyer reaction of hippuric acid or 4-methoxy hippuric acid with indole-3-carboxaldehyde **2** in acetic anhydride and fused sodium acetate. Interestingly, the IR spectra of compounds **9a,b** explored two (C = O) stretching bands for each at 1634, 1689 and 1788, 1743 cm^−1^, respectively, assigned for the *N*-acetyl and oxazolone carbonyl groups, respectively. This confirmed that the (NH) group of the indole ring was acetylated under the reaction conditions. ^1^H NMR of both compounds revealed a singlet signal at 1.90 ppm assigned for the (CH_3_) protons of the acetyl moiety. Subsequent reaction of compounds **9a,b** with the appropriate sulfonamide derivatives **3a–f** afforded the expected imidazolinone derivatives **10a–l** through a mechanism involving an open intermediate. The IR spectra of compounds **10a–l** showed two (C = O) bands in the range of 1730–1635 cm^−1^ and 1701–1597 cm^−1^ and two characteristic bands for the (SO_2_) group at 1338–1300 and 1168–1126 cm^−1^. ^1^H NMR demonstrated a singlet signal in the range of 1.90–2.08 ppm assigned for the CH_3_ protons of the acetyl group, in addition to the (OCH**_3_**) group protons in compounds **10 g–l** in the range of 3.80–3.81 ppm. Moreover, compounds **10 b** and **10 h** revealed an additional singlet signal at 1.92 and 2.04 ppm, respectively, assigned for (CH_3_) protons of the acetamido sulfamoyl moiety. ^13^C NMR spectra of this series were in accordance with their carbon skeleton where the (CH_3_) group of the indole acetyl moiety appeared in the range of 24.0–24.4 ppm, in addition to the (CH_3_) signals of the second acetamide moiety at 23.6 and 22.0 ppm in compounds **10 b** and **10 h**, respectively. Also, compound **10 h** was characterised by another signal corresponding to the (OCH_3_) group at 55.7 ppm.

### Carbonic anhydrase inhibitory activity

The CA inhibitory activities of compounds **4a–f**, **7a–f**, and **10a–l** in addition to acetazolamide (AAZ) as standard inhibitor, were measured against hCAI, hCA II, hCA IV and hCA IX by a stopped flow CO_2_ hydrase assay^29^. The rational for the choice of these four isoforms is that hCA II and IV are targets for antiglaucoma drugs^1–3^, whereas hCA IX have been validated as targets for the treatment and prognosis of hypoxic cancers^39^^,^^40^. Otherwise hCA I is one of the main off-target isoforms both for the antiglaucoma or anticancer CAIs therapeutic application^1^^,^^3^.

The following structure-activity-relationships (SARs) were obtained from the inhibition data reported in Table 1.

**Table 1.** Inhibition data of human CA isoforms hCA I, II, IV and IX with compounds **4a–f**, **7a–f** and **10a–l** reported here and the standard sulfonamide inhibitor acetazolamide (**AAZ**) by a stopped flow CO_2_ hydrase assay.

	K_I_^a^ (nM)			
Compound	hCA I	hCA II	hCA IV	hCA IX
**4a**	88.5	575.8	2744.6	327.4
**4 b**	8576.7	>10000	>10000	1615.9
**4c**	>10000	>10000	>10000	>10000
**4d**	>10000	>10000	>10000	>10000
**4e**	>10000	>10000	>10000	>10000
**4f**	7534.5	8288.3	>10000	1314.5
**7a**	96.4	76.4	4429.3	308.0
**7 b**	7978.0	>10000	>10000	778.3
**7c**	>10000	>10000	>10000	>10000
**7d**	>10000	>10000	>10000	>10000
**7e**	>10000	>10000	>10000	>10000
**7f**	9067.9	7181.8	>10000	1396.7
**10a**	96.6	56.4	262.7	318.4
**10 b**	5867.4	487.3	>10000	1467.2
**10c**	>10000	>10000	>10000	>10000
**10d**	>10000	>10000	>10000	>10000
**10e**	>10000	>10000	>10000	>10000
**10f**	5764.1	3816.2	>10000	1636.4
**10 g**	93.7	602.3	2771.2	328.0
**10 h**	5719.1	548.9	3048.1	1400.3
**10i**	>10000	>10000	>10000	>10000
**10j**	>10000	>10000	>10000	>10000
**10k**	>10000	>10000	>10000	>10000
**10 l**	6898.2	>10000	>10000	1206.6
**AAZ**	250	12	74	25

^a^Mean from three different assays, by a stopped flow technique (errors were in the range of ±5–10% of the reported values).

(i) As expected the inhibitory profile of the tested compounds against the four CA isoforms was strictly dependent on the functionalisation mode of the sulfonamide group, with the primary SO_2_NH_2_ bearing derivatives (**4a**, **7a**, **10a,** and **10 g**) arisen as the best hCA inhibitors herein reported. Sulfonylacetamido (**4 b**, **7 b**, **10 b,** and **10 h**) and sulfonylguanidino (**4c**, **7c**, **10c,** and **10i**) compounds maintained a certain degree of activity, dependent on the tailing moiety and the considered isozymes. Conversely, a totally counterproductive effect on the inhibition potency can be ascribed to all other *N*^1^-functionalisations, with all following considerations regarding uniquely the active ZBG bearing-derivatives.

(ii) The cytosolic isoform hCA I was significantly inhibited by all primary sulfonamide compounds with K_I_s in the range of 88.5–96.6 nM. It is pertinent to mention that the corresponding sulfonylguanidine (**10f**) and sulfonylacetamide (**10 h**) derivatives lose to a great extent their efficacy, showing a K_I_ of 5764.1 and of 5719.1 nM, respectively.

(iii) hCA II was the most inhibited isoform, among those considered here by the primary sulfonamide derivatives (**4a**, **7a**, **10a,** and **10 g**) with K_I_s value 575.8, 76.4, 56.4, 602.3 nM, respectively. In analogy to the SAR reported for the previous isoform, it is interesting to note that the nature of the tail appended at the benzenesulfonamide scaffold greatly affected the inhibition of this isoform in the sulfonylacetamide subset, being the imidazolones (**10 b**) and (**10 h**) medium nanomolar inhibitors (K_I_ 487.3 and 548.9 nM), and imine (**4 b**) and thiazolinone (**7 b**) devoid of any efficacy.

(iv) Uniquely the primary sulfonamides (**4a**, **7a**, **10a,** and **10 g**) exhibited significant activity against the membrane-associated isoform IV (K_I_s spanning between 262.7 and 4429.3 nM), with exception of sulfonylacetamide (**10 h**) which showed a micromolar inhibition with a K_I_ of 3048.1 nM.

(v) The general tendencies described above were also applicable to the tumour-associated isoform hCA IX, which was moderately inhibited by the primary sulfonamides (**4a**, **7a**, **10a,** and **10 g)** with binding affinities ranging between 308.0 and 328.0 nM. Sulfonylacetamido (**4 b**, **7 b**, **10 b,** and **10 h)** and sulfonylguanidino (**4c**, **7c**, **10c,** and **10i**) compounds were shown to possess comparable effects on hCA IX, inhibiting it in the low micromolar range (K_I_s of 778.3–1615.9 nM and 1206.6–1636.4 nM).

(vi) The inhibition data reported in Table 1 unexpectedly highlighted a total loss of efficacy upon functionalisation of the ZBG primary sulfonamide structure by pyridine, thiazolinone and pyrimidine moieties, at least within the present set of imine-, thiazolidinone- and imidazolone-indole benzenesulfamides. On the other hand, incorporation of less hindered groups as in case of sulfonylacetamides and sulfonylguanidines maintained a certain degree of activity dependent on the tailing moiety and the considered isozymes.

(vii) Comparing the results of the current study with the earlier investigation in which a chromone nucleus was used instead of the indole ring^21^, revealed that the chromone derivatives showed excellent activity profile despite being secondary sulfonamides and bearing similar *N*^1^-substitutions. So, it can be claimed that activity is not solely linked to the absence or presence of the *N*^1^-substitution, but also affected by other distant fragments in the molecule (chromone *vs*. indole). In other words, the presence of secondary sulfonamide moiety is not to be exclusively accused of the dramatic decrease in activity of the indole derivatives compared to their chromone counterparts.

## Conclusions

Since the primary sulfonamide is the most efficient zinc binding group (ZBG) to design inhibitors for the metallo-enzymes CAs, herein, we propose an investigation on four physiologically important human (h) CAs (I, II, IV, and IX) of *N*^1^-substituted secondary sulfonamides incorporating a thiazolinone or imidazolone-indole scaffold. The effect of functionalisation of the sulfonamide group with five different substitution patterns, namely acetyl, pyridine, thiazole, pyrimidine and carbamimidoyl, was evaluated in relation to the inhibition profile of the primary sulfonamide analogues. With most of these latter compounds acting as nanomolar inhibitors of all four considered isoforms, a total loss of inhibitory efficacy upon functionalisation of the ZBG primary sulfonamide structure arose in case of incorporation of pyridine, thiazole and pyrimidine moieties. On the other hand, incorporation of less hindered groups, such as sulfonylacetamide (**4 b**, **7 b**, **10 b,** and **10 h**) and sulfonylguanidino (**4c, 7c**, **10c,** and **10i**) maintained a certain degree of activity dependent on the tailing moiety and the considered isozymes, with K_I_s spanning in the low micromolar range. Nevertheless, it is worth to be mentioned that the same functionalisation of the sulfonamide group but with chromone scaffolds, led to potent inhibitors, in the nanomolar range as revealed in a previous report^21^. This drew our attention to the role of the indole nucleus as a key fragment responsible for switching the activity of this type of compounds.
